# Immunological recovery in tuberculosis/HIV co-infected patients on antiretroviral therapy: implication for tuberculosis preventive therapy

**DOI:** 10.1186/s12879-017-2627-y

**Published:** 2017-07-25

**Authors:** Basel Karo, Gérard Krause, Stefanie Castell, Christian Kollan, Osamah Hamouda, Walter Haas, the ClinSurv HIV Study Group, A. Kühne, K. Arastéh, F. Bergmann, M. Warncke, J. Rockstroh, J. Wasmuth, S. Hass, B. Jensen, C. Feind, S. Esser, P. Schenk-Westkamp, C. Stephan, A. Haberl, P. Schott, A. Plettenberg, F. Kuhlendahl, H.-J. Stellbrink, A. Adam, K. Schewe, S. Fenske, T. Buhk, C. Hoffmann, D. Radzuweit, A. Mainka, O. Degen, M. Franz, N. Treffler, M. Stoll, R. Beider, S. Gerschmann, K. Hoeper, H.A. Horst, S. Trautmann, G. Fätkenheuer, D. Gillor, J. Bogner, B. Sonntag, B. Salzberger, C. Fritzsche

**Affiliations:** 1 Department for Infectious Disease Epidemiology, Robert Koch Institute (RKI), Seestr. 10, 13353 Berlin, Germany; 2 PhD Programme, “Epidemiology”, Braunschweig-Hannover, Germany; 3 Department of Epidemiology, Helmholtz Centre for Infection Research (HZI), Braunschweig, Germany; 4 Hannover Medical School (MHH), Hannover, Germany

**Keywords:** HIV/aids, Tuberculosis, Antiretroviral therapy, Immune recovery, Developed country

## Abstract

**Background:**

Understanding the immune response to combination antiretroviral therapy (cART) is essential for a clear approach to tuberculosis (TB) preventive therapy. We investigated the immunological recovery in cART-treated HIV-infected patients developing TB compared to those who remained free of TB.

**Methods:**

We extracted data of HIV-infected patients from a multicenter cohort for the HIV clinical surveillance in Germany. No patients included in our study had TB at the beginning of the observation. Using a longitudinal mixed model, we assessed the differences in the mean change of biomarkers (CD4+ cell count, CD8+ cell count, CD4:CD8 ratio and viral load) since cART initiation in patients who remained free of TB vs. those developing TB. To detect the best-fit trajectories of the immunological biomarkers, we applied a multivariable fractional polynomials model.

**Results:**

We analyzed a total of 10,671 HIV-infected patients including 139 patients who developed TB during follow-up. The highest TB incidences were observed during the first two years since cART initiation (0.32 and 0.50 per 100 person-years). In an adjusted multivariable mixed model, we found that the average change in CD4+ cell count recovery was significantly greater by 33 cells/μl in patients who remained free of TB compared with those developing TB. After the initial three months of cART, 65.6% of patients who remaining free of TB achieved CD4+ count of ≥400 cells/μl, while only 11.3% of patients developing TB reached this immunological status after the three months of cART. We found no differences in the average change of CD8+ cell count, CD4:CD8 ratio or viral load between the two-patient groups.

**Conclusion:**

All HIV-infected patients responded to cART. However, patients developing TB showed reduced recovery in CD4+ cell count and this might partly explain the incident TB in HIV-infected patients receiving cART. These findings reinforce the importance of adjunctive TB preventive therapy for patients with reduced recovery in CD4+ cell count.

## Background

Infection with HIV is the most potent risk factor for tuberculosis (TB) [1]. HIV causes functional and numeric depletion of TB-specific lymphocytes, leading to impairment of both cell-mediated immune response, and granuloma formulation and maintenance [2]. Therefore, co-infection with HIV increases the risk of progression to active TB, and reactivation of latent TB infection (LTBI) [3]. The introduction of combination antiretroviral therapy (cART) in the mid-1990s has markedly reduced HIV-related morbidity, and mortality [4]. Although cART is associated with a dramatic decline in TB risk by 70%-90% among HIV-infected patients [5], TB incidence remains elevated in settings with high [5, 6] and low TB incidence [5, 7]. Empirical observations and modelling studies suggests that cART may have limited long-term impact on TB incidence at a population level [8]. Also, HIV infection was associated with lower TB treatment success rates even in settings with available cART [9, 10].

TB preventive therapy can significantly reduce the risk of TB among HIV-infected patients, even among those receiving cART, and living in areas with low TB rates [11, 12]. Recently, TB preventive therapy for high-risk groups including HIV-infected patients has attracted increasing attention in developed countries, as WHO launched a new action framework towards TB elimination in low incidence countries [13]. It is recommended that preventive therapy should be offered to HIV-positive patients particularly for those with positive tuberculin skin tests (TST) [11, 12]. Such an approach has limitations due to the low sensitivity of TST among HIV-infected patients [14]. It has been observed that the proportion of TST-positive results is associated with higher CD4+ cell count, and TST-negative HIV-infected patients could still develop active TB [15, 16]. The greater the immunosuppression, the lower the probability of positive skin reaction [14, 17]. This means that there is an increased risk that HIV-infected patients most in need will be left without preventive therapy. Similarly, interferon-gamma release assays (IGRAs) have clearly low sensitivity in HIV-positive patients and do not offer any advantage over TST [18]. A multicenter European study (TBNET Study) showed that progression towards active TB in immunocompromised patients was poorly predicted by TST or IGRAs [19].

The majority of studies defined HIV-infected patients at high risk for TB and, therefore, those who might benefit from preventive therapy based on baseline CD4+ cell count [6, 7, 20]. In addition to the lack of consensus on the optimal threshold, this approach does not take into account that TB risk changes over time in association with ART-induced immune recovery. Despite the advances in HIV literature on the immune response to cART, there is still limited information on immune recovery in relation to TB development. Investigating the growth dynamic of immune biomarkers under cART is essential to understand the risk of TB development in cART-treated patients and helps to improve our TB preventive therapy approach.

In this study, we aimed to investigate whether the average change in immunological biomarkers is associated with TB development and to analyze the immunological dynamic in cART-treated HIV-infected patients in relation to development of active TB.

## Methods

### Data source and study population

The study was based on the German ClinSurv HIV Cohort, which is an ongoing open multicentre cohort for the clinical surveillance of HIV in Germany. The ClinSurv HIV cohort was established in 1999 as a collaborative cohort between specialized treatment centres for HIV/AIDS and the Robert Koch Institute. Irrespective of the disease stage, all HIV-infected patients are eligible to attend one of the treatment centres and can be enrolled in the ClinSurv HIV Cohort. The ClinSurv dataset includes basic demographic data recorded anonymously at the first contact. Clinical and laboratory data are collected and updated in 3-month-periods when data are available. The German ClinSurv HIV Cohort has been described in detail elsewhere [7, 21].

For our analysis, we included patients who started cART between 1999 and 2013. cART was defined as “triple-drug therapy” involving two nucleoside reverse-transcriptase inhibitors (NRTI) plus one non-nucleoside reverse-transcriptase inhibitor (NNRTI) or protease inhibitor. We excluded patients who developed TB during the first 3 months after cART initiation. These cases may reflect unmasking active TB known as TB-associated immune reconstruction inflammatory syndrome (TB-IRIS) [22]. These cases are not clinically apparent prior to cART initiation and manifest after the recovery of the immune system induced by HIV therapy [22]. TB cases were defined as any form of TB bacteriologically confirmed or clinically diagnosed.

No patient included in our analysis has received TB preventive therapy during follow-up observation. No data were available on LTBI testing in the German HIV Cohort.

### Statistical analysis and modeling approach

Continuous variables were described using medians, and interquartile ranges (IQR) and compared by a nonparametric K-sample test on the equality of medians. Categorical variables were described using absolute and relative frequencies and compared by the χ^2^ test for difference in proportion.

We used a Cox proportional hazards regression model (survival analysis) to estimate the TB incidence rate defined as the number of TB cases occurring per 100 person-years of observation. The observation period was calculated as the time from cART initiation to either diagnosis of TB, last follow-up, a maximum of a 7-year observation period (very few measurements were available beyond seven years of follow-up) or the last possible observation date (30 June 2014).

We investigated the difference in the mean change of each laboratory measurements (CD4+ cell count, CD8+ cell count, CD4:CD8 ratio, and viral load) overtime on cART between patients remained free of TB vs. those developing TB. Laboratory measurements were coded as time-varying variables and were the main outcome variables. Subjects included in the analysis required at least two serial measurements; measurements taken after TB diagnosis were not included in the analysis. Baseline measurements were defined as the measurements taken at the date of cART initiation. We fitted a separate longitudinal mixed model for each laboratory biomarkers with change since cART initiation at one year interval. The purpose of this approach is to investigate the pattern of the average change in biomarkers over time on cART. We adjusted the models for sex, geographical origin, HIV transmission routes, age, year of cART initiation and baseline value of biomarkers (CD4+ cell count, CD8+ cell count, and viral load). The models were corrected with a random intercept and random slope to account for both individual differences in the average change in immune recovery and individual differences in trajectories across the time of the study. A variance-covariance structure was specified to account for the potential correlation between repeated measurements for the same individuals. Moreover, we added an interaction term between TB and time on cART to compare the trajectory of change between patients remained free of TB with those developing TB.

To analyze the growth dynamics of the laboratory biomarkers over time on cART, we used multivariable fractional polynomials (MFP) to account for nonlinear association and to detect the best-fit model [23]. The MFP approach was adjusted for sex, geographical origin, HIV transmission routes, age, year of cART initiation and involves three steps: (1) test of covariates inclusion by backward elimination at a *p*-value of <0.1, (2) test of non-linearity at a *p* value of <0.05, (3) test of simplicity of power transformation (first degree FP vs. second degree FP) at a *p*-value of <0.05. Parameters in the final model were estimated using the generalized estimating equations (GEE) method. We used the GEE method to account for possible correlation among the repeated measurements taken in an individual and to deal with longitudinal data on subjects with unequally spaced and varying number of observations (accounting for missing data). All analyses were performed using STATA (version 14, StataCorp, LP, Texas, USA).

## Results

### Characteristics of the study population

**Fig. 1:**
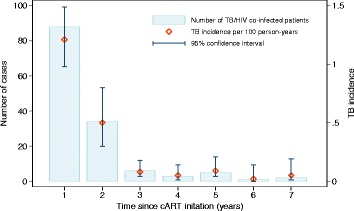
Tuberculosis incidence among HIV-infected patients since cART initiation in the German ClinSurv HIV cohort, 1999-2013. TB cases diagnosed within the first three months of cART initiation were excluded. cART: combination antiretroviral therapy

From January 1999 through December 2013, a total of 11,293 HIV-infected patients initiated cART in the German ClinSurv HIV Cohort. Patients known to have TB prior to cART (*N* = 371), diagnosed with TB within the initial 3 months of cART (*N* = 86), or with less than two available serial laboratory measurements (165) were excluded from our analysis. The majority of HIV-infected patients remaining in our study (*N* = 10,671) were male (77.4%), originated from Germany (69.8%), and had a median age of 38 years at the time of cART initiation. The baseline median CD4+ count was 285 cells/μl [IQR 172 – 413], the baseline median CD8+ count was 896 cells/μl [IQR 603 – 1301], the baseline median CD4:CD8 ratio was 0.3 [IQR 0.2 – 0.5], and the baseline viral load was 4.6 log_10_ copies/ml [IQR 3.6 – 5.2]. A total of 139 (1.3%) patients developed TB during 43,854 person-years follow-up (incidence of 0.32 per 100 person-years of follow-up; 95% confidence interval (CI) 0.27-0.37). Higher incidences were observed during the first two years since cART initiation (1.2 and 0.5 per 100 person-years respectively) (Fig. 1). Differences in demographic and clinical characteristic between patients who remained free of TB and those developing TB are presented in Table 1.

**Table 1:** Characteristic of cART-treated HIV-infected patients in the German ClinSurv HIV Cohort, 1999-2013

Demographic and clinical characteristic of HIV patients, *N* = 10,671 (100%)	HIV-infected patients who remained free of TB, *N* = 10,532 (98.7%)	HIV-infected patients developing TB, *N* = 139 (1.3%)	*P* value
Female sex	2388 (22.5%)	45 (31.5%)	0.01^b^
Age at time of cART initiation			
Median (IQR), years	38.0 (31 – 45)	36.5 (31 – 45)	0.8^c^
Geographical origin			
Germany	7289 (70.1%)	60 (42.9%)	<0.001^b^
Sub-Saharan Africa	1344 (12.9%)	50 (35.7%)	
South−/Southeast Asia	273 (2.7%)	10 (7.1%)	
Others	1486 (14.3%)	20 (14.3%)	
HIV-transmission routes			
MSM	5377 (56.9%)	49 (36.6%)	<0.001^b^
IDUs	886 (9.4%)	8 (6.0%)	
Others	3186 (33.7%)	77 (57.4%)	
Calendar year of cART initiation			<0.001^b^
1999-2002	2524 (23.9%)	56 (24.2%)	
2003-2008	4531 (43.1%)	58 (43.0%)	
2009-2013	3477 (33.0%)	25 (32.8%)	
Baseline CD4+ count^a^			
Median (IQR), cells per μl	285 (173 – 413)	168 (80 - 326)	0.1^c^
Baseline CD8+ count^a^			
Median (IQR), cells per μl	900 (602 – 1297)	846 (609 – 1364)	0.8^c^
Baseline CD4:CD8 ratio^a^			
Median (IQR)	0.3 (0.2 – 0.5)	0.2 (0.1 – 0.3)	0.3^c^
Baseline viral load^a^			
Median (IQR), log_10_ copies per ml	4.6 (3.6 – 5.2)	4.9 (3.7 – 5.1)	0.06^c^

*cART* combination antiretroviral therapy, *TB* tuberculosis, *IQR* interquartile range, *MSM* men who have sex with men, *IDU* injecting drug users

^a^Baseline was defined as the measurements taken at the date of cART initiation

^b^Obtained using a χ^2^ test for difference in proportion

^c^Obtained using a non-parametric K-sample test on the equality of medians

### Trend of change in biomarkers since cART initiation in relation to tuberculosis

**Fig. 2:**
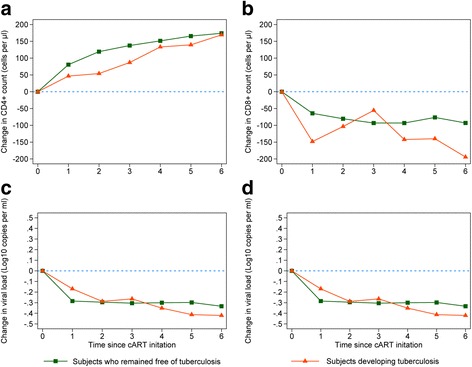
Change in biomarkers since cART initiation among HIV-infected patients in the ClinSurv HIV cohort, 1999-2013. (**a**) Change in CD4+ cell count. (**b**) Change in CD8+ cell count. (**c**) Change in CD4:CD8 ratio. (**d**) Change in viral load

In a mixed model with adjustment for sex, geographical origin, HIV transmission routes, age, year of cART initiation and baseline value of biomarkers (CD4+ cell count, CD8+ cell count, and viral load), the recovery of biomarkers over time on cART (increasing in CD4+ cell count and CD4:CD8 ratio; decreasing in CD8+ cell count and viral load) was significant for all investigated biomarkers and irrespective to TB (Table 2) (Fig. 2). However, patients remaining free of TB had significantly a more marked increase in the change of CD4+ cell count since cART initiation than those developing TB. No significant difference in the pattern of change for CD8+ cell count, CD4:CD8 ratio or viral load was observed between the two-patient groups (Table 2) (Fig. 2).

**Table 2:** Change in biomarkers since cART initiation among HIV-infected patients in the ClinSurv HIV cohort, 1999-2013

Biomarkers	Estimated change over time on cART (per year)	*P* value
CD4+ count	43.9 cells per μl (95% CI 43.1 to 44.6)	<0.001
CD8+ count	−27.9 cells per μl (95% CI −28.6 to −27.2)	<0.001
CD4:CD8 ratio	0.06 (95% 0.06 to 0.07)	<0.001
Viral load	−0.1 log_10_ copies per ml (95% CI −0.1 to −0.1)	<0.001
Biomarkers	Estimated mean of difference in change of biomarkers between cART-treated patients developing TB vs. those who remained free of TB	*P* value
CD4+ count	−33 cells per μl (95% CI −44.6 to 21.2)	0.001
CD8+ count	−72 cells per μl (95% CI −84.6 to 1.7)	0.1
CD4:CD8 ratio	−0.01 (95% CI −0.03 to 0.01)	0.2
Viral load	0.01 log_10_ copies per ml (−0.04 to 0 .06)	0.7

*cART* combination antiretroviral therapy, *TB* tuberculosis, *IQR* interquartile range

Longitudinal mixed models adjusted for sex, geographical origin, HIV transmission routes, age, year of cART initiation and baseline value of biomarkers (CD4+ count, CD8+ count, and viral load) and including interaction term between time and TB

### Growth dynamics of CD4+, CD8+, CD4:CD8 ratio, and viral load

**Fig. 3:**
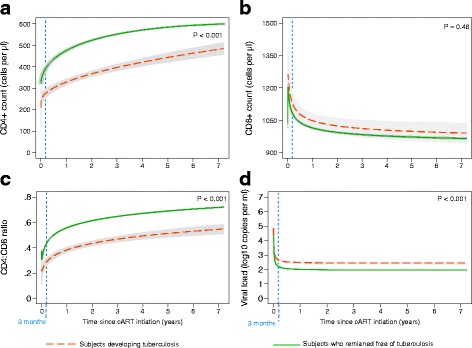
Immunological recovery since cART initiation among HIV-infected patients in the German ClinSurv HIV Cohort, 1999-2013. (**a**) Profile plot of CD4+ cell count. (**b**) Profile plot of CD8+ cell count. (**c**) Profile plot of CD4:CD8 ratio. (**d**) Profile plot of viral load. The best-fitting model of CD4+ cells count is FP2 (0, 0.5) for subjects who remained free of TB and is FP2 (−1,1) for subjects who developed TB. Irrespective to TB development, the best-fitting model of CD8+ cells count is FP2 (−0.5, −0.5)], of CD4:CD8 ratio is FP2 (−0.5, −0.5), and of viral load is FP2 (−2, −0.5). No measurements were included after TB diagnosis. TB: tuberculosis; FP2: second-degree fractional polynomial model

After cART initiation, the general pattern of the CD4+ cell count was increasing over time but at a decreasing rate. Compared to patients remaining free of TB, patients developing TB showed less marked increase in CD4+ cell count over time (*P* < 0.001) (Fig. 3a). After the initial 3 months of cART, 65.6% of patients who remaining free of TB achieved CD4+ count of >400 cells/μl, while just 11.3% of patients developing TB reached this status within the same period (corresponding to 60% vs. 25% increase in CD4 cell counts compared to the baseline values respectively). Similarly, the general pattern of CD4:CD8 ratio improved substantially over time; again patients who remained free of TB showed faster recovery compared to patients developing TB (*P* < 0.001) (Fig. 3c). After the initial phase, patients remaining free of TB reached on average a 0.44 CD4:CD8 ratio, which was achieved by 56.1% of them vs. 8.3% of patients developing TB.

In the initial 3 months of cART, CD8+ cell count showed a sharp rise followed by a sharp decline reaching approximately 1100 cell/μl after 3 months of cART. Afterwards, CD8+ cell count decreased slowly over time. No significant difference was observed in the dynamics of CD8+ cell count in relation to development of active TB (*P* = 0.48) (Fig. 2b). HIV viral load showed a sharp drop in the initial phase of cART reaching approximately 2.5 log_10_ copies/ml followed by a plateau (Fig. 3d). Patients who remained free of TB had a plateau at a significantly lower level compared to those developing TB (Fig. 3d). We applied piecewise-linear trend models to assess the non-linearity and patterns of change of biomarkers and it confirmed our findings and showed no significant differences from the trajectories obtained by the MPF models (data not shown).

### The trend of CD4+ cell count close to the time of tuberculosis diagnosis

Using a fractional polynomial fit, we observed a decline in CD4+ cell count around 5 months before TB diagnosis. However, this pattern in CD4+ cell count did not show a significant drop since the fractional polynomial growth does not fit the data significantly better than the linear fit (deviance difference = 0.89; *P* = 0.8). In the linear regression model adjusted for sex, geographical origin, HIV-transmission routes, age, and year of cART initiation, CD4+ cell count continued to recover over time irrespective of the time of TB diagnosis [RC 0.07 CI 0.004 - 0.14, *P* = 0.04]. We could not investigate how the trend of viral load, CD8+ cell count, and CD4:CD8 ratio developed at the time close to TB diagnosis as there were insufficient measurements of these variables around the time of TB diagnosis.

## Discussion

We investigated the immunological recovery in cART-treated HIV-infected patients in the German HIV cohort in relation to development of active TB. Although patients developing TB showed effective response to cART, we found that the average change of increases in CD4+ count was significantly smaller by 33 cells/μl compared with patients who remained free of TB. After the initial 3 months of cART, 65.6% of patients who remaining free of TB achieved CD4+ count of >400 cells/μl, while just 11.3% of patients developing TB reached this status within the same period. We found no differences in the change of CD8+ cell count, CD4:CD8 ratio or viral load between the two-patient groups.

Excess TB rates during early cART were observed in many studies and were attributed to cART-induced unmasking of subclinical TB [7, 24]. However, we still found a higher TB incidence in the first year of cART in our study population, even after excluding cases that occurred in the first 3 months of cART. The mechanisms underlying development of TB in this period are not fully understood and reflect a spectrum of complex interactions between mycobacterial antigen load and cART-induced immune response [22, 25–27]. Given the risk of subsequent transmission due to unrecognized active TB and the high disease burden of unmasked TB, all possible efforts are needed to prevent it. Early initiation of cART before significant immune depletion will potentially decrease the risk of unmasking TB [26, 27].

In our data, patients developing TB started cART at lower CD4+ cell count compared to patients remaining free of TB. Despite not being statistically significant, this lower baseline count may reflect a greater degree of pre-treatment immunodeficiency, requiring for patients developing TB a more prolonged treatment period for CD4+ cell count to recover [28] and therefore could partially explain the reduced recovery among them. Different studies done in high and low income countries showed that baseline CD4+ cell count was independently associated with higher risk of TB [6, 7, 20]. However, the increase of CD4+ cell count remained significantly greater in patients who remained free of TB after controlling for baseline biomarkers. This different increase in CD4+ cell count might partially explain the risk of TB in cART-treated HIV-infected patients. These results are supported by findings from a current study that showed that bacterial infections in HIV-infected people was associated with change in CD+ cell count since study entry [29]. Other studies showed that baseline CD4+ cell count did not have any predictive value for TB risk after controlling for the updated CD4+ cell count [30, 31].

The overall recovery of CD4+ cell count in the first 3 months of cART was relatively rapid, and then continued to rise at a slower rate. This is consistent with other findings from HIV studies that show rapid increases in CD4+ cell count among cART-treated patients predominantly occur in the first months with comparatively smaller gains thereafter [32–35]. Importantly, patients developing TB lacked comparable immune recovery and spent a substantial time at lower CD4+ cell count and showed smaller increase in CD4+ count. Therefore they remained at high TB risk for a long period of time and this was mirrored by the higher TB incidence occurring during this period. Immunological studies suggest that full restoration of lymphocytes only occurs in a minority of cART-treated patients and long-term recovery of TB-specific immune function remains incomplete despite continuous suppression of HIV replication [36, 37]. This hinders the extent to which cART can contribute to TB control [37].

Isoniazid preventive therapy (IPT) is the most widely used regimen and is strongly recommended by WHO to decrease the burden of TB in HIV-infected patients. TB preventive therapy can significantly reduce the risk of TB among HIV-infected patients, even among those receiving cART, and living in areas with low TB rates. However, implementation of IPT at the same time as ART may be problematic due to the difficulty in excluding active TB at baseline and unmasking TB during the first months of ART. Furthermore, inadequate patient adherence to IPT and fear of promoting resistance to isoniazid through IPT represent other major barriers to IPT implementation [38]. Different studies have suggested the use of preventive therapy not only at baseline but also serially or continuously for patients at higher risk of TB [29, 39]. Our data add strong evidence to use immune recovery to identify patients who could benefit from adjunctive TB preventive therapy even if they initially respond to cART. In light of our findings, a logical approach might be to wait 3 months after cART initiation in order to exclude unmasking TB and then consider TB preventive therapy for patients with reduced immune recovery. A prospective study is required to confirm these findings and to identify a threshold of CD4+ cell count blow which preventive therapy should be considered in comparison to a LTBI testing approach.

Similar to CD4+ cell count, CD4:CD8 ratio in patients developing TB relatively recovered at a lower rate. Other studies have shown that despite effective cART and CD4+ recovery above 500 cells/μl, a persistently low CD4:CD8 ratio acts as a marker of continuing immune dysfunction [40], and the onset of HIV-related diseases [41]. However, controlling for baseline biomarkers revealed no significant difference in the average change of CD4:CD8 ratio over time on cART.

We found that irrespective of TB progression, CD8+ cells count increased markedly in the initial weeks of cART, followed by a significant decrease and subsequent plateau for the rest of follow-up. Elevation of CD8+ cell count occurs in the very early days of HIV infection as a general reaction to viral infections [42]. Sharp falls in CD8+ cell count can be observed shortly after the initiation of cART but CD8+ cell count remain persistently elevated during HIV infection and do not normalize despite long-term cART [42, 43]. It is well-known that CD8+ cells play an important role in the elimination of both TB- and HIV-infected cells and consequent immune control. Dysmature CD8+ cells are a common phenomenon in HIV-infected patients; these CD8+ cells produce significantly lower levels of perforin, granulysin, and TNF-α which are crucial components of a protective immune response to TB infection [42]. Again these facts highlight the limitation of cART in TB prevention in HIV-infected patients.

HIV viral load plateaued rapidly after a steep decline in the initial 3 months of cART in both patients developing TB and those who remained free of TB. However, a higher level of viral load persisted among patients developing TB compared to those who remained free of TB. This might reflect the differences in growth dynamic of CD4+ cell count between them, where restoration of CD4+ cell count is associated with the degree of suppression of HIV replication.

Interestingly, our data showed a decline in CD4+ cell count close to the time of TB diagnosis (around 5 months prior). However, this finding was not statistically significant and needs to be repeated with a larger sample size in order to investigate the usefulness of a drop in CD4+ cell count in predicting the onset of active TB. This hypothesis is supported by the fact that depletion of CD4+ cell count impairs cell-mediated immune responses, and granuloma formation and function leading to an increased risk of active TB [2].

There are some limitations to this study. Our analyses involved relatively few TB events (*n* = 139), which might limits the study power. We could not investigate the trend of CD8+ cell count, and viral load close to TB diagnosis due to missing data. It is unknown whether TB diagnosis was systematically performed among all HIV-infected patients in the ClinSurv HIV Cohort (active case finding) or whether it was just a part of on-going health care (passive case finding); therefore some TB cases may not have been detected. The success rate of and adherence to cART may play role in the immunological response and it was not included in our analysis due to lack of data. However, current study on the ClinSurv HIV cohort showed that around 95% of the patients in the cohort achieve viral suppression (viral load < 50 copies/ml), suggesting a high success rate and adherence to cART among patients [44].

## Conclusion

Our findings highlight the strong association between TB and the cART-induced response of CD4+ cell count in HIV-infected patients. Although the initially response to cART was observed, patients developing TB showed reduced cART-induced increases in CD4+ cell count. This might partly explain the incident TB in HIV-infected patients receiving cART. These findings reinforce the importance of adjunctive TB preventive therapy for those patients with reduced recovery in CD4+ cell count. Prospective studies are needed to confirm protective immunological thresholds for TB with an adequate assessment of cART adherence.

## Abbreviations

cART: Combination antiretroviral therapy
GEE: Generalized estimating equations.
IQR: Interquartile ranges
MFP: Multivariable fractional polynomial
NNRTI: Non-nucleoside reverse-transcriptase inhibitor
NRTI: Nucleoside reverse-transcriptase inhibitors
TB: Tuberculosis
TB-IRIS: TB-associated immune reconstruction inflammatory syndrome
TST: Tuberculin skin test
